# The role of *t* test in beer brewing

**DOI:** 10.3325/cmj.2020.61.69

**Published:** 2020-02

**Authors:** Vladimir Trkulja, Pero Hrabač

**Affiliations:** 1 Department of Pharmacology, Zagreb University School of Medicine, Zagreb, Croatia *vtrkulja@mef.hr*; 2 Department of Medical Statistics, Epidemiology, and Medical Informatics, “Andrija Štampar” School of Public Health, University of Zagreb School of Medicine, Zagreb, Croatia

## The Student's *t* test

In previous columns, we touched on certain concepts in statistics that form the basis of statistical thinking. In this text, we will deviate briefly from the general concepts and focus on a single statistical test. Namely, we will discuss the basics of the *t* test, known also as the Student's *t* test, one of the basic tests in statistics, which besides everyday applicability has a very interesting history.

## The man behind the test

The Student's test was named (obviously) after a man known as “Student,” although his real name was William Sealy Gosset, born on 13 June 1876 in Canterbury, Kent, England. Much earlier, at the end of the 14th century, Canterbury was immortalized in The Canterbury Tales by G. Chaucer. Other notable citizens included W. Harvey, the first physician to give a precise and detailed description of human systemic circulation (1). As the oldest child in the family of an English Army colonel, Gosset received very good education at Winchester College, an institution with a tradition longer than 500 years. Although he intended to follow in his father’s footsteps, he was turned down by the military due to poor eyesight. Instead, he opted for plan B – the study of chemistry at Oxford, where he graduated with First Class degree in 1899. Immediately after his studies, he took a job as a brewery master at the Guinness Brewery, where he remained until his death in 1937.

At the beginning of the 20th century, Guinness started hiring Oxford and Cambridge graduates to introduce scientific methods into beer production, and Gosset proved to be an excellent choice. Already in 1904 he presented a plan to the brewery supervisory board that included the application of the law of error to improve the production process. During his whole working life, Gosset was in constant written contact with other contemporary statisticians, particularly Karl Pearson (father), Egon Pearson (son), and R.A. Fisher, but unfortunately much correspondence was later lost. Gosset also attended Karl Pearson lectures at University College in London, although he commented (somewhat disappointedly) that his knowledge of mathematics was not good enough for him to benefit from these lectures. On the other hand, his meeting with K. Pearson in the summer of 1905 was one of the crucial events in Gosset’s career. He later referred to this meeting as a half an hour in which Pearson introduced him to almost all the statistical methods known at the time. Armed with such knowledge, Gosset embarked upon a task of introducing new methodology to the brewery (2).

## Theory and application

The theoretical basis K. Pearson transmitted to Gosset was based on the 18th and 19th century learning, particularly on the works of Bayes, Gauss, and Laplace. The methodology for calculation of probability of events, normal distribution, and least squares method, and foundations of the central limit theorem were already well known, but in order to be applicable, these methods required a relatively large number of data. On the other hand, problems encountered by brewers, such as the impact of fermentation temperature on the acidity of beer, have mainly resulted in a small number of measurements, often fewer than 5. Gosset describes this issue in his first work, published in 1907 in *Biometrika*, which he wrote under the pseudonym “Student.” The pseudonym was invented by the then director of Guinness, following the company's policy that employees may publish the results of their work under two conditions – not to mention the company in any context and not to publish under their real names. Thus, Gosset is remembered in history as “Student.”

Being familiar with the central limit theorem, Gosset assumed that the observed values of beer acidity – as well as their possible differences (for example – under different fermentation conditions) – would follow Gauss's (normal) distribution. The normal distribution is defined by two parameters: mean and standard deviation. Although both values can be calculated even when the number of observations is very low, eg, 3 or 5, standard deviation obtained from such a small sample is quite unreliable. Therefore, the problem was how to describe the distribution of the observed beer acidity values if we have too few measurements to calculate the standard deviation with sufficient accuracy. In addition, since Gosset was interested in the acidity difference between the two groups, we are actually talking about the (theoretical sampling) distribution of the values of differences between groups and, consequently, the standard deviation of these differences.

To solve this problem, Gosset developed a method of approximation of standard deviation when the number of observations is very small. The theoretical distribution calculated from such approximated data did not correspond to the normal distribution. Searching for a distribution that would resemble the normal distribution but for a smaller number of observations, Gosset actually rediscovered something that German geodetic engineer F.R. Helmert described much earlier, ironically just in the year of Gosset's birth (1876) (3). Since the works of German mathematicians were not known in England at the time, Gosset considered the discovery of this distribution as his own achievement and called it a t-distribution. Unlike the normal distribution, the t-distribution is closely related to the number of observations (ie, degrees of freedom), so its shape changes according to the number of measurements. Therefore, the t-distribution belongs to the family of symmetric distributions and takes different shapes depending on sample size, ie, degrees of freedom (dfs). Actually, the t-distribution starts to resemble the normal distribution as the number of observations increases (especially over 30), while for a smaller number of observations, the t-distribution is lower and wider than the normal distribution (ie, it is flatter at the peak and “thicker” at the tails).

## The *t* test (for independent samples)

This test is applied when one wants to test an *a priori* hypothesis (eg, the null hypothesis) about differences in mean values of a certain outcome between two independent samples (groups) of observational units. A version of the test exists also when the tested hypothesis pertains to repeatedly measured values in the same observational units (*t* test for paired data), but here we will discuss the more common application.

Clearly, the outcome is quantified on an interval scale (ie, the outcome variable is a continuous variable), and the effect measure is the difference in mean values. The difference in means is an outcome measure that has a sampling t-distribution (with the respective dfs). The essence of the test is to estimate the ratio of the average (mean) difference between the two samples (groups) to the intra-group variability of the measured outcome, ie, to estimate whether the two means differ beyond the intra-group variability of the measured outcome. This ratio is called the t-value, ie, the t-value is the test statistic. It is actually the standardized difference between the two means (the difference divided by its standard error).

Although the formula for calculating the t-value may seem complex to non-mathematicians, one needs only a basic school level of mathematics to understand it.

Let us assume that the question of interest was whether one-month-old laboratory rats of two different strains (strain 1 and strain 2), housed and cared for under identical conditions, differed in body weight. Such a question actually defines an *a priori* statistical hypothesis – the null hypothesis. It says that the difference in body weight between one-month-old members of the two strains is 0. The *t* test is conducted to assess whether the observed data (results of the actual measurement) are compatible or not with such a hypothesis. There are no assumptions about into which direction the difference could go (ie, whether body weight is higher in strain 1 or in strain 2).

In a random sample of 5 animals from strain 1, the following values are measured: 137, 135, 136, 138, 142 g.

In a random sample of 5 animals from strain 2, the following values are measured: 124, 126, 131, 126, 141 g.

The first step is to calculate the mean body weight for each sample: 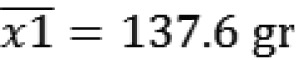 and 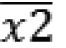 = 129.2 gr. Based on this calculation alone, it appears that the animals form strain 1 are on average heavier. However, by observing only the mean values, we do not get a clear picture about the dispersion of the measurements. Therefore, in the next step, standard deviations (SD) in the two samples are calculated: SD1 = 2.70 and SD2 = 6.05 g. The test statistic, t-value, is then simply calculated based on the two mean values, two SDs, and the number of animals per group:


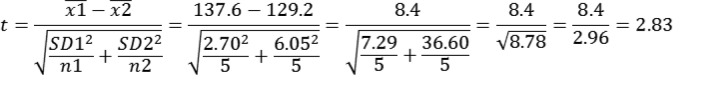


The calculated t-value suggests that the between-group difference (difference between two mean values) is approximately 2.8 times greater than the variability of the observations within the two groups. This suggests that the two samples come from two populations that substantially differ in this characteristic (weight of animals), ie, that the two strains differ in this respect.

The above calculation process actually comprises two steps. The first step is to estimate the parameter of interest (the difference between two strains in this characteristic) – which is a simple difference between mean values. The next step is to estimate the standard error of this estimate (the standard deviation of the respective sampling t-distribution of this effect measure). Therefore, the same equation given above can be divided into two steps: the first step is the calculation of the pooled (or joint) SD for the two samples (pooled SD, SDp):


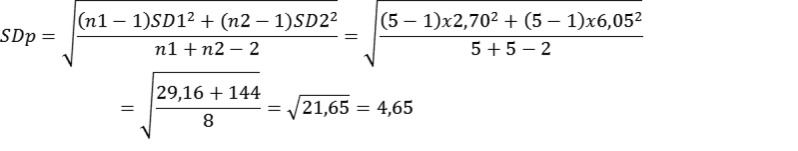


The second step – using SDp to calculate the standard error (SE) of the difference (SEd):


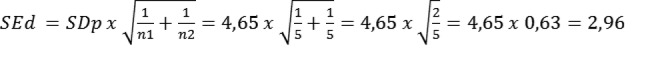


Then, the t-score is the ratio of the observed difference (x1-x2) and SEd:


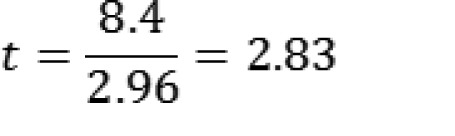


As any frequentist hypothesis test, the *t* test ends with the generation of a *P* value – an index that quantifies the level of compatibility of the observed effect with the *a priori* hypothesis. In this case, the null hypothesis is that difference strain 1 - strain 2 = 0. As elaborated in our previous articles (4,5), low *P* values indicate the incompatibility of the observed effect with the *a priori* hypothesis, while high *P* values indicate compatibility with the null hypothesis (although there has been a wide debate about the use and interpretation of *P* values).

Here, we explain only the process of determination of the *P* value that corresponds to a certain t-value (ie, test statistic generated in an independent *t* test). The process of the *P* value generation in this test consists of a few steps: (i) we establish to which percentile of the sampling t-distribution with 8 dfs the calculated t-value fall. In this specific case, the t-distribution has 8 dfs, since the total sample size was 10 (n1+n2 = 5 + 5 = 10), but 1 df was “used” to calculate the mean value in the sample 1 and one was used to calculate the mean value in the sample 2, hence 10-2 = 8 values that are “free to vary” (8 dfs); (ii) we calculate the area under the curve of the respective t-distribution between this percentile and the 100th percentile. This area is called the “*P* value.” Finally, since the null hypothesis is a two-sided hypothesis (makes no assumptions about the direction into which the difference could go) – this area is multiplied by 2 to yield the two-sided *P* value. If the t-value is -2.83, the process is the same, except that the area between the respective percentile and 0 is determined. In this specific case, the two-sided *P* value = 0.022. While today the calculation is done by statistical software, in Gosset’s time the researchers needed to use the so-called t-tables (one can use them today as well – they are available online). These tables contain critical information about the range of the t-distributions, ie, t-distributions with a wide range of dfs. For each given distribution, they contain the t-values that correspond to certain “critical” percentiles of such a distribution. For example, if one accepts the *a priori* criterion that 5% of the values in the respective sampling distribution are “extremes” (ie, t-values equal to or higher than the t-value corresponding to the 97.5th percentile and t-values equal to or lower than the t-value corresponding to the 2.5th percentile, ie, a total of 5% of the values) – then the t-value corresponding to these percentiles is shown. In the current case, the calculated t-value is 2.83. In a table of t-distributions, one needs to find a t-distribution with 8 dfs and read the value of the t-score that corresponds to the 97.5th percentile of such a distribution, which is 2.365. Hence the current t-value is higher than the one corresponding to the 97.5th percentile – and, hence, the current t-value falls within the “5% of the extremes.” The exact area under the curve “above” the calculated t-score (2.83) of an 8-df t-distribution equals 0.011, and when it is multiplied by 2 it gives a two-sided *P* value of 0.022.

This value tells us that the observed data are not really compatible with the null hypothesis, ie, they do not support it. Conventionally, we say that the null hypothesis should be rejected, ie, the data are more in favor of a conclusion that the difference between the two strains is 8.4 g (or greater) and not 0. The *P* value, however, does not tell us “how much more” it is in favor of this conclusion (vs the null hypothesis). It says that if you repeat the entire process in the exactly the same way an infinite number of times, and if the null hypothesis is true, you will observe the effect of this size (or a more extreme one) only 2.2% of the time. For a probabilistic statement about the support to the null or to the alternative, one needs a Bayesian approach and calculation of the Bayesian “analogue,” ie, the Bayes factor.
